# Reproductive Declines in an Endangered Seabird: Cause for Concern or Signs of Conservation Success?

**DOI:** 10.1371/journal.pone.0019489

**Published:** 2011-05-03

**Authors:** Justin Schuetz

**Affiliations:** Audubon California, Emeryville, California, United States of America; Texas A&M University, United States of America

## Abstract

Collection and analysis of demographic data play a critical role in monitoring and management of endangered taxa. I analyzed long-term clutch size and fledgling productivity data for California least tern (*Sternula antillarum browni*), a federally endangered subspecies that has recently become a candidate for down-listing. While the breeding population grew from approximately 1,253 to 7,241 pairs (578%) during the study period (1988–2009) both clutch size and fledgling productivity declined. Clutch size decreased by approximately 0.27 eggs (14%) from 1990–2004 then showed a moderate increase of 0.11 eggs from 2004–2009. Estimates of fledgling productivity showed a similar pattern of decline and moderate increase even after controlling for clutch size. Sea surface temperature anomalies, an index of El Niño-Southern Oscillation activity, did not influence clutch size but were associated with fledgling productivity through a non-linear relationship. Both clutch size and fledgling productivity increased with latitude, potentially indicating a gradient of life-history trade-offs. Random site effects explained little of the overall variation in clutch size (3%) or fledgling productivity (<1%) suggesting that site characteristics beyond those associated with latitude had little bearing on either measure of reproduction. Despite intensive monitoring and management, causes of variation in key demographic parameters remain poorly understood. Long-term declines in clutch size and fledgling productivity may reflect: 1) reduced food availability, 2) increased density-dependent competition, and/or 3) age-dependent reproduction coupled with a shifting population age-structure. Until the mechanisms shaping demographic parameters and population change are better understood, the success of past management and the probability of ongoing recovery will remain difficult to characterize.

## Introduction

Successful management of endangered species depends critically on understanding the ecological and evolutionary forces that shape population dynamics. When collected and analyzed carefully, demographic data, including rates of survival and reproduction, may help researchers to identify the key mechanisms driving population change [1]. They may also guide management of the specific life stages that have the greatest potential for affecting population growth and persistence [2]. Here, I describe patterns of variation in clutch size and fledgling productivity in the federally endangered California least tern (*Sternula antillarum browni*) in an effort to develop consensus about the subspecies' biology and needs with respect to future recovery planning and assessment.

Historically, California least terns bred in colonies containing thousands of birds but by 1973 the state breeding population consisted of only 624 pairs [3]. Causes of the population decline are difficult to determine because few historical population estimates exist. Even so, loss of breeding habitat to coastal development and disturbance of nesting birds by humans and dogs were identified as potential problems in the early 1900 s [4], [5]. Unlike other species of terns, California least terns apparently did not suffer large-scale harvest for the millinery trade [6]. As a result of its small population size and perceived risk of extinction, the subspecies was listed as endangered by US Fish and Wildlife (USFWS) in 1970 (Federal Register 35: 8491, June 2, 1970).

**Figure 1:**
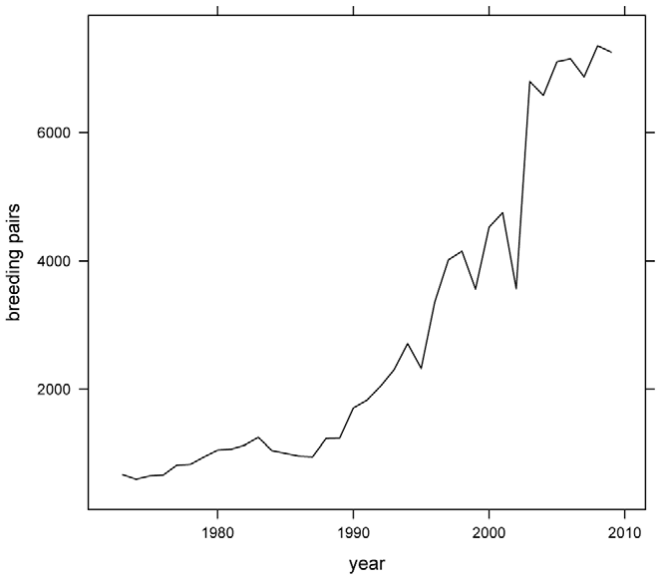
Number of California least tern pairs breeding in California from 1973–2009.

The USFWS developed a recovery plan to guide conservation and recovery efforts that established three targets for delisting: 1) increase the breeding population to 1200 pairs in California; 2) establish at least 20 pairs in each of 20 “secure” management areas (with at least 4 “secure” areas in San Francisco Bay Area; 6 in Mission Bay; and 6 in San Diego Bay); and 3) realize a mean reproductive rate over 5 years of 1.0 young fledged per pair [3]. Management activities, including fencing colonies and controlling predators, were implemented at many sites throughout the state and are largely credited with increasing the state breeding population to more than 7000 pairs [7] (Figure 1). In 2006, USFWS conducted a 5-year review of the status of California least tern and recommended down-listing the subspecies from a status of endangered to threatened although only one of three recovery goals has been met (i.e., number of breeding pairs) [6]. The San Francisco Bay Area has yet to harbor 4 “secure” colonies and estimates of fledgling production have rarely approached 1.0 young per pair in recent years [6].

To better understand the biology of the subspecies and the processes that have shaped population growth, I explored patterns of temporal and spatial variation in clutch size and fledgling productivity. To characterize temporal variation I examined trends across years and in relation to the El Niño-Southern Oscillation (ENSO), an irregular (3–7 year) climate cycle in which sea surface temperatures (SST) fluctuate between periods of relatively warm (El Niño) and cool (La Niña) conditions. I focused on SST as a potential driver of clutch size and fledgling productivity because researchers have attributed small clutch sizes [8] and poor productivity [9] at California least tern nesting sites to reduced food availability associated with warm El Niño conditions. I expected annual variation in clutch size and fledgling productivity to be negatively associated with SST. To characterize spatial variation I examined latitudinal trends. I expected that clutch size would increase with latitude, as it does for many bird species [10], [11], and that fledgling productivity would increase correspondingly.

## Methods

### Study species

The California least tern (*Sternula antillarum browni*) is a small-bodied (∼45 g), migratory seabird that nests along the Pacific coast of North America. It is one of three subspecies of least tern that occur in North America and the breeding population is distributed discontinuously from San Francisco Bay to Baja California [6]. After returning from their wintering grounds in mid- to late April, birds establish breeding colonies on bare or sparsely vegetated beaches, dunes, or gravelly areas often in close proximity to rivers, estuaries, or lagoons. Most females begin breeding in their third year of life [12] and lay two spotted beige eggs in a shell- or debris-lined depression in the sand. Semi-precocial young hatch after 19–25 days of incubation and fledge approximately three weeks later [13]. California least terns are relatively long-lived. Adult survivorship estimates range from 0.84–0.94 [9], [13], [14] and banded birds have been recovered after 15 years [15].

### Data sets

**Figure 2:**
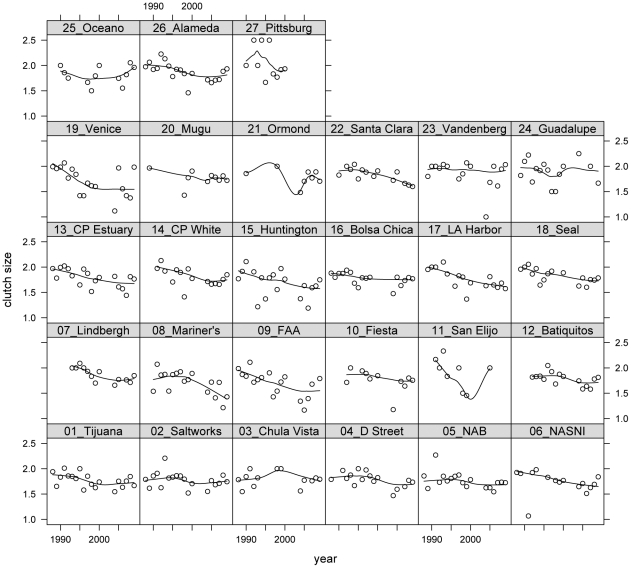
Plots of clutch size by year conditional on site. Sites are ordered by latitude with Tijuana Estuary being the southernmost site and Pittsburg Power Plant being the northernmost. Dots indicate clutch size estimates in a single season and lines are smoothers used to reveal patterns in the data.

I assembled data on California least tern population size, clutch size, and fledgling production at breeding sites throughout California using California Department of Fish and Game (CDFG) breeding season surveys. Typically, data were reported at the level of the “site” but at some sites data were partitioned to the level of “colony” or “sub-colony”. I lumped partitioned data if colonies or sub-colonies were geographically indistinct or if colony and sub-colony data were not separated in every year. I split data at sites if colonies or sub-colonies were geographically distinct and data were reported consistently. My intention was to capture the biology of the birds on as fine a geographic scale as possible given differences among sites and years in data reporting. Site classifications are available upon request. I used data from 27 sites for which more than five years of clutch size and productivity data were available between 1988–2009 (Figures 2–4). These sites contained an average of 97% of the breeding pairs during that period.

**Figure 3 pone-0019489-g003:**
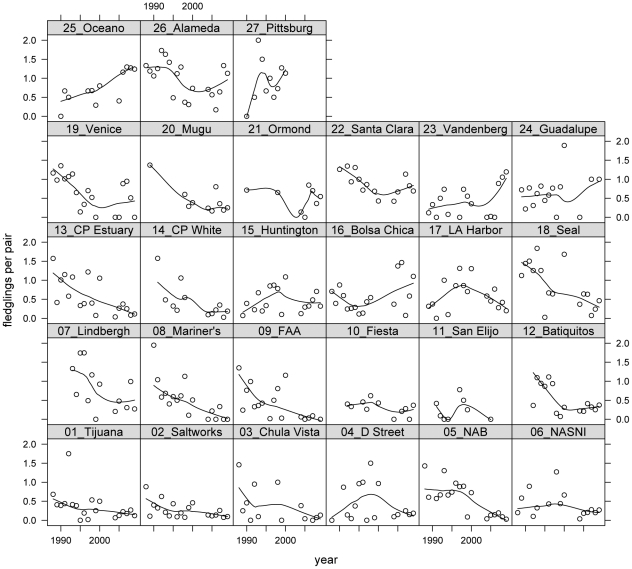
Plots of fledgling productivity by year conditional on site. Sites are ordered by latitude with Tijuana Estuary being the southernmost site and Pittsburg Power Plant being the northernmost. Dots indicate estimates of fledglings per pair in a single season and lines are smoothers used to reveal patterns in the data.

**Figure 4:**
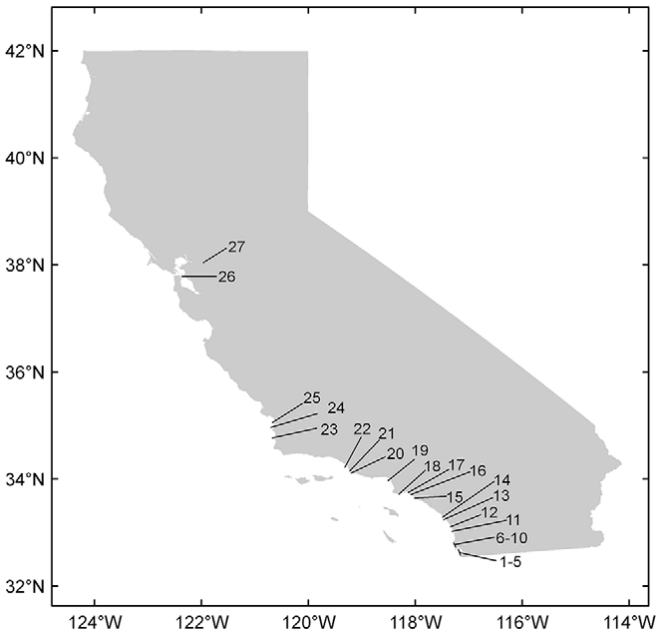
California least tern breeding sites used in this study. Site numbers correspond to those in Figures 2 and 3.

### Response variables

#### Clutch size

Clutch size data represent an average for each site. Because monitors were typically unable to follow individually marked pairs throughout the entire breeding season, site averages include data from first clutches and from clutches laid to replace depredated or abandoned nests.

#### Fledgling productivity

I calculated fledgling productivity (fledglings per pair) using fledgling numbers at each site and the total number of breeding pairs at each site. When monitors provided minimum and maximum estimates of fledgling or pair numbers at sites I used the average. Both fledgling and pair numbers can be difficult to estimate because young are hard to follow until fledging and adults are rarely individually identifiable unless captured. Several techniques for counting fledglings and breeding pairs have been used by monitors and are described by Marschalek [7]. I assumed that estimates provided by monitors were unbiased across years and sites and were suitable for providing perspective on long-term, large-scale patterns.

### Covariates

#### Year

I included year as a continuous covariate in all analyses to assess whether there have been significant trends in clutch size or fledgling productivity since 1988.

#### Sea Surface Temperature (SST) Anomaly

I characterized ocean conditions using SST anomalies described in the Extended Reconstruction Sea Surface Temperature dataset (ERSST.v3b) [16] as reported by the National Weather Service Climate Prediction Center [17]. I used the three-month running mean SST anomalies in the Niño 3.4 region (5°N–5°S, 120°–170°W). Anomalies were calculated by the Climate Prediction Center using 1971–2000 as the base period for comparison [18]. California least terns typically begin nesting in mid- to late May, so I used the April–May–June anomaly (SST-AMJ) as a predictor in the analysis of clutch size and the June–July–August anomaly (SST-JJA) as a predictor in the analysis of fledgling productivity.

#### Latitude

I included latitude as a covariate in all analyses. In California, the breeding range of California least terns extends from the Tijuana River Estuary (32.56° N) to the northern edge of San Francisco Bay (38.19° N).

#### Clutch size

I included clutch size as a covariate in the analysis of fledgling productivity to determine whether variables in addition to clutch size influenced fledgling production.

### Analysis of clutch size

After characterizing the distribution of clutch sizes and plotting relationships between clutch size and each of the covariates, I attempted to fit a linear mixed regression model to the data in the lme4 package [19] in R [20]. Year, SST-AMJ, and latitude were specified as covariates and site was included as a random intercept. Residuals from the model showed marked patterning when plotted against year, violating the assumption of homogeneity. As a result, I adopted a generalized additive mixed modeling approach [21]. I specified site as a random effect and used splines to model the effects of year, SST-AMJ, and latitude in the mgcv package [22]. I performed model selection using backward stepwise elimination.

### Analysis of fledgling productivity

Numbers of fledglings produced at study sites in each year were non-negative counts. I fit a general linear mixed regression model to the data in the lme4 package and specified a Poisson distribution for the errors. I accounted for the different numbers of pairs at each site by including the natural logarithm of breeding pairs as an offset in the regression model. I specified year, SST-JJA, latitude, and clutch size as covariates and site as a random effect. There was marked patterning of the residuals, particularly when plotted against clutch size, so I adopted a generalized additive mixed modeling approach using the mgcv package [23]. I specified site as a random effect and used splines to model the effects of year, SST-JJA, latitude, and clutch size. I performed model selection using backward stepwise elimination. R code for both analyses is available upon request.

## Results

### Clutch size

**Figure 5:**
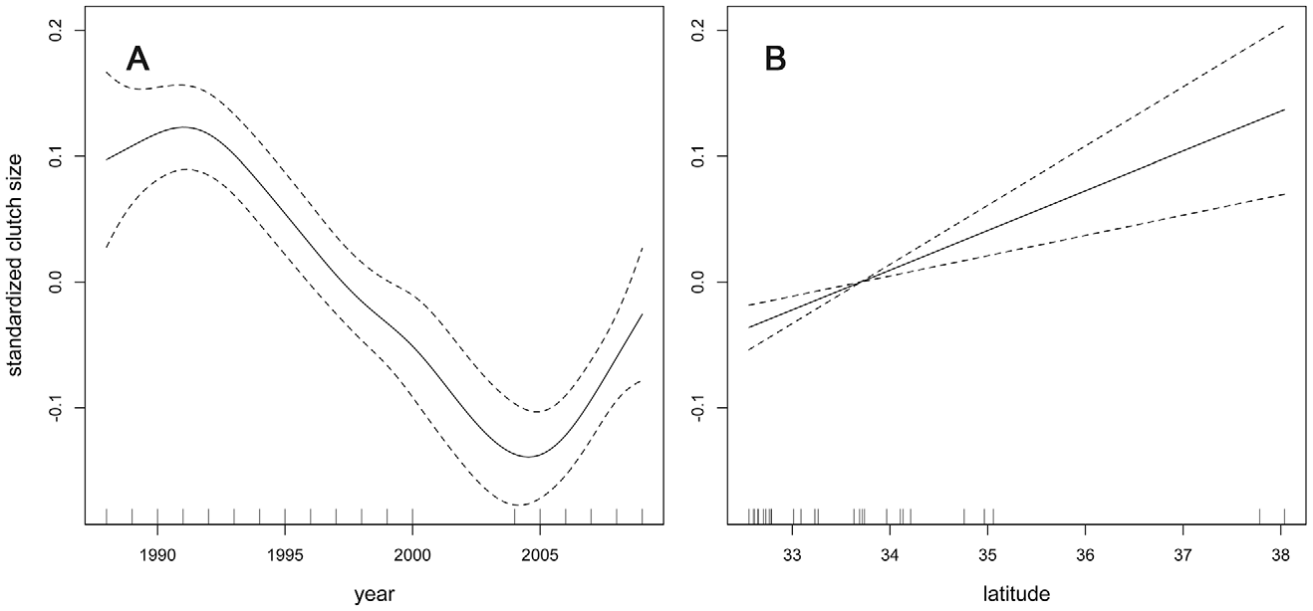
Partial dependence plots showing marginal effect of A) year and B) latitude from analysis of clutch size. The solid lines show fits derived from the generalized additive mixed model. Dotted lines indicate 95% confidence intervals. Clutch size data were unavailable from 2001–2003.

Clutch size showed a significant non-linear pattern over time (Figure 5A, p<0.0001), peaking at 1.92 eggs in 1990, decreasing by approximately 14% to 1.65 eggs in 2004, and subsequently increasing to 1.76 eggs in 2009. Latitude was also a significant predictor (p<0.0001) of clutch size. Clutch size increased by more than 0.03 eggs with every degree of latitude, resulting in a predicted difference of approximately 0.17 eggs between the southern and northern extremes of the California breeding range (Figure 5B). Counter to expectation, SST anomalies for the three-month period spanning April–May–June were not associated with variation in clutch size (p = 0.42) and the covariate was dropped from the final model (Table 1). Differences among sites, which were modeled with a random intercept, explained only a small fraction (∼3%) of the total variance in clutch size.

**Table 1:** Results of final generalized additive mixed model for clutch size.

Parametric coefficients:				
	estimate	SE	t	p
intercept	0.72	0.26	2.77	<0.01
latitude	0.03	0.01	4.09	<0.0001

Approximate significance of smooth term:			
	estimated df	F	p
s(year)	4.64	22.98	<0.0001

R^2^(adjusted) = 0.25.

n = 404.

Latitude was originally fit as a smooth term but is reported as a parametric coefficient below because the relationship was linear.

### Fledgling productivity

**Figure 6:**
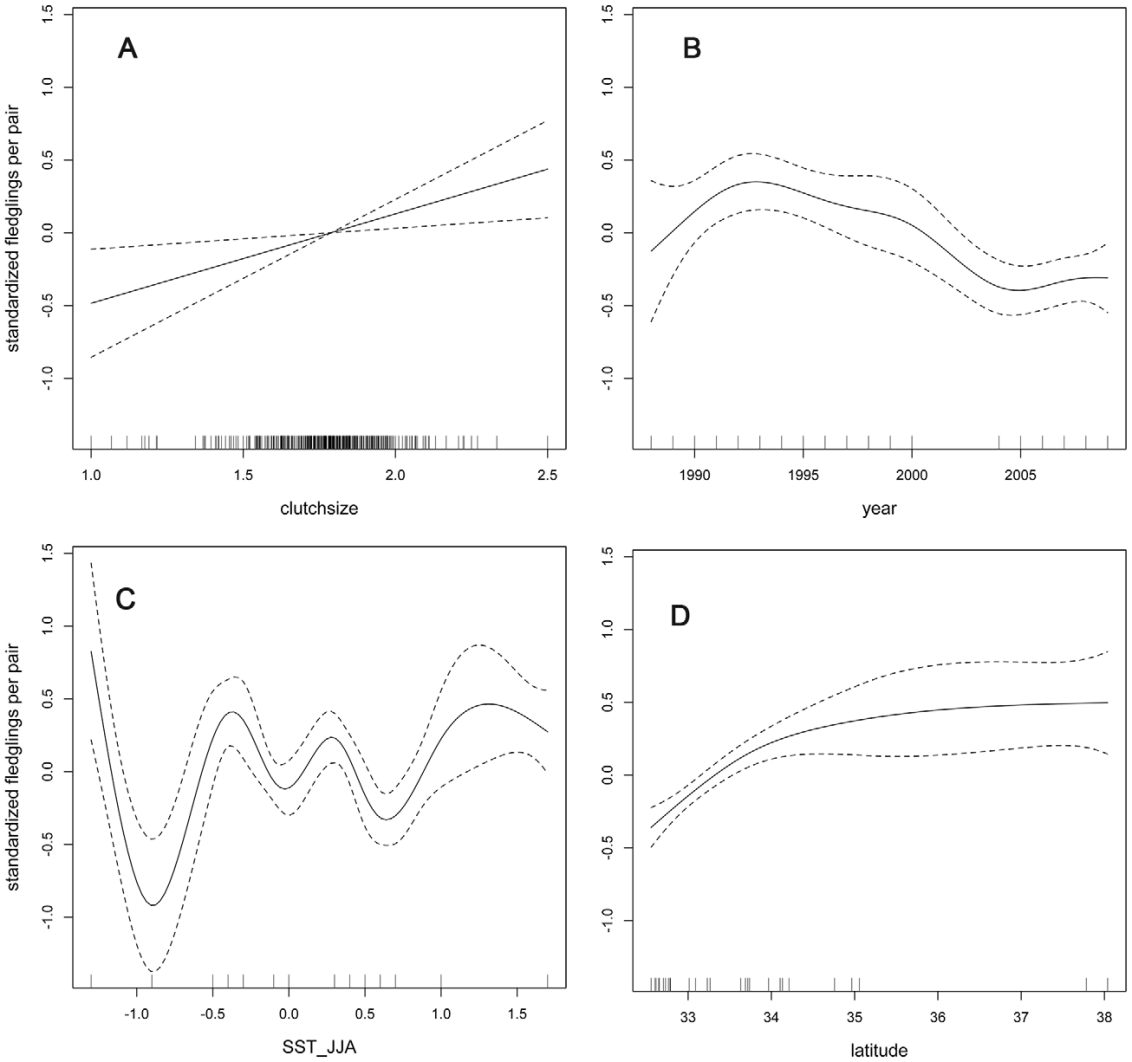
Partial dependence plots showing marginal effect of A) clutch size, B) year, C) SST-JJA, and D) latitude from analysis of fledgling productivity. The solid lines show fits derived from the generalized additive mixed model. Dotted lines indicate 95% confidence intervals. Clutch size data were unavailable from 2001–2003, precluding analysis of productivity in those years.

As expected, fledgling productivity was positively associated with clutch size (Figure 6A, p<0.01, Table 2). At the mean clutch size (1.79 eggs), California least terns fledged 0.50 young. At two standard deviations above and below the mean (1.38 and 2.20 eggs), terns fledged 0.39 and 0.65 young. Fledgling productivity decreased inconsistently (Figure 6B) from 1988–2009. Birds were most efficient at converting eggs to fledglings in 1991 (0.95 fledglings per pair) and least efficient in 1999 and 2004 (0.23 fledglings per pair in both years). SST anomalies for the three-month period spanning June–July–August appeared to significantly affect variation in fledgling productivity though the shape of the relationship was complicated (Figure 6C, p<0.0001). Latitude was a significant predictor of productivity (Figure 6D, p<0.0001) with northern birds fledging young more efficiently than southern birds, even while controlling for larger clutches in the north. Random site effects explained a negligible amount of the variation in productivity (<1%) suggesting that site characteristics, beyond those captured by latitude, had little bearing on productivity throughout the range of California least tern.

**Table 2:** Results of final generalized additive mixed model for fledgling productivity.

Parametric coefficients:				
	estimate	SE	t	p
intercept	−1.81	0.42	−4.26	<0.0001
clutch size	0.63	0.23	2.66	<0.01

Approximate significance of smooth terms:			
	estimated df	F	p
s(year)	3.78	12.90	<0.0001
s(latitude)	2.17	13.45	<0.0001
s(SST_JJA)	7.86	7.96	<0.0001

R^2^(adjusted) = 0.70.

n = 401.

Clutch size was originally modeled as a smooth term but is reported as a parametric coefficient below because the relationship was linear.

## Discussion

From a recovery viewpoint, the most compelling result to emerge from my analyses was the decline in estimates of clutch size and fledgling productivity between 1990 and 2004. While the breeding population increased more than 350% (1828 to 6580 breeding pairs) average clutch size decreased by 14% (1.92 to 1.65 eggs) and birds became less efficient at hatching and/or rearing young even while controlling for decreasing clutch size. The similar shapes of clutch size and productivity trends, with peaks in the early 1990 s and troughs in 2004, suggest that similar ecological or demographic forces have influenced both breeding parameters. Among numerous possible explanations three stand out, each suggesting very different interpretations of the success of past management and prospects for lasting recovery.

### Hypothesis 1: Food limitation due to shrinking prey base

Terns collect the resources necessary for reproduction on the breeding grounds [24] and their breeding success may be influenced by prey availability [25]. Assuming the same is true for California least terns, the clutch size and productivity declines described here may have resulted from decreasing prey availability throughout the California Current. Unfortunately, there are few data available to assess whether there has been a widespread and long-term decline in prey availability that coincides with the patterns I detected. Chavez et al. [26] described a series of multi-decadal shifts in Pacific Ocean conditions that resulted in alternating warm “sardine regimes” and cool “anchovy regimes”. Interestingly, the pattern of sardine landings mirrors the trends in tern clutch size and productivity with a peak around 1990 followed by a consistent decline to 2001 (the last year for which they reported data). There is little evidence to suggest a direct causal link between sardine abundance and tern reproduction, however. In fact, California least tern diets differ markedly among sites [8] and years [27] and there is some indication that productivity is correlated with anchovy abundance [28], not sardines. Assessing whether (and how) food has limited tern reproduction will require significant effort. An index of prey availability will only be useful if it can account for the breadth and flexibility of California least tern diets across the range of the subspecies.

### Hypothesis 2: Food limitation due to increasing density-dependent competition

Patterns of increasing population size accompanied by decreasing clutch size and productivity suggest the possibility of population regulation through increasing density-dependent competition [29], [30]. During seabird breeding seasons, a halo of locally depleted prey can emerge around colonies [31] that causes birds to search for food at greater distances as the season progresses or as colony size increases [32], [33]. Several studies of seabirds suggest intraspecific competition does increase with colony size, and that adult condition, provisioning rates, chick survival, and per capita reproduction decrease as a result [32], [34], [35].

Competition for local resources may constrain California least tern clutch size and productivity particularly because they forage near breeding colonies. Atwood and Minsky [36] observed foraging behavior of California least terns at three sites and noted that birds foraged in nearshore waters within 4 km of nesting sites. If density-dependent competition drove the declines I describe, both clutch size and fledgling productivity should be negatively correlated with the number of pairs breeding at a site (i.e., colony size). In post hoc analyses, I did not detect any effect of colony size on clutch size. However, fledgling productivity did appear to decline at colonies harboring more than 1000 pairs suggesting a potential effect of density-dependent competition at a small number of large breeding sites.

### Hypothesis 3: By-product of age-dependent reproduction coupled with a shifting population age-structure

Long-term trends in clutch size and fledgling productivity may also emerge as a by-product of age-dependent variation in clutch size and productivity combined with a changing population age-structure. In the closely related common tern (*Sterna hirundo*), older individuals tend to lay larger clutches than younger individuals [37]. In addition, productivity improves with age [38], potentially as a consequence of more efficient chick provisioning [39] with more energy-rich prey [40]. If California least terns show similar patterns of age-dependent reproduction, then the declines in clutch size and productivity I detected may simply reflect successful management. As relatively large numbers of fledglings were produced and subsequently recruited to the breeding population after the implementation of predator control in the 1980 s, the average age of the population would have declined, driving down average clutch size and fledgling productivity. Similarly, increases in clutch size and productivity seen since 2005 may have resulted from a slowly aging population that has received relatively few recruits in recent years.

Despite extensive banding at some breeding sites, there is no published information on age-dependent reproduction in California least tern and there are few estimates of population age-structure. Focused banding and recapture efforts, particularly at sites with historic age-structure estimates (e.g., Camp Pendleton [41] and Venice Beach [9]) could be used to establish whether clutch size and productivity increase with age and to compare historic and current age-structures. Intensive banding efforts at one or a small number of sites might also allow researchers to develop less labor-intensive methods for assessing age. After calibrating methods with known-age birds, long-term monitoring of population age-structure could potentially be achieved using telomeres [42], [43] (but see [44]).

### ENSO cycles

Counter to expectation, I did not find evidence for a clear link between SST and clutch size or fledgling productivity in California least terns. The apparent lack of a SST effect on clutch size may indicate one or more of the following: 1) clutch size investment is relatively immune to environmental influence, 2) SST measured in the Niño 3.4 region is a poor predictor of SST at California least tern breeding sites, 3) SST is a poor predictor of breeding conditions for terns, and/or 4) the biological consequences of SST anomalies do not emerge until several months after their measurement. The last explanation seems unlikely. I performed a series of post hoc analyses using SST anomaly data from earlier three-month periods and they did not improve the model fit. Interestingly, Atwood and Kelly [8] documented reduced clutch size in 1982 at the very beginning of an El Niño but not in 1983 soon after some of the warmest SST anomalies on record were recorded.

In contrast, productivity was significantly associated with SST anomalies for the three-month period spanning June–July–August. The relationship was complicated, however, (Figure 6C) and did not imply a straightforward mechanism linking ocean temperature and breeding success. In an effort to simplify the relationship, I performed a post hoc analysis in which I treated SST-JJA as a linear predictor. While the model provided a poorer fit to the data, productivity was significantly positively associated with SST-JJA, suggesting that birds were more efficient at rearing young in warm-water conditions. My finding differs from that of Massey et al. [9] who documented significant mortality of birds around Venice Beach in association with the 1982–1983 El Niño warming event and a persistent dent in the age-structure of the local population due to lack of recruitment. While predictions of the frequency, intensity, and duration of El Niño conditions in the future remain highly uncertain [45], there are likely to be consequences of periodic SST anomalies and climate change for California least terns. My analyses suggest that the mechanistic links between water temperature anomalies and tern biology will only be revealed by incorporating more detailed oceanographic data into models.

### Geographic considerations

The data and analyses reveal marked patterns of geographic variation in clutch size and productivity. Clutch size increased by approximately 0.03 eggs with every degree of latitude. Latitudinal variation in clutch size is common in birds, including terns, though the mechanisms that determine the pattern have long been debated [46]. Regardless of the cause, a marked gradient in clutch size suggests that opportunities for, and constraints on, recovery may differ across the range of California least tern due to fundamental differences in life-history strategies. My analysis of fledgling productivity also indicated that parenting efficiency (i.e., the ability to convert eggs to fledglings) was significantly associated with latitude. Northern populations of terns hatched and/or reared young more efficiently than southern populations. The difference could arise as a consequence of latitudinal differences in food availability, weather, methods for estimating fledgling numbers, and/or life-history trade-offs. The last alternative is particularly intriguing from an evolutionary ecology perspective and implies that birds in northern populations invest relatively more in reproduction than birds in southern populations, potentially at the cost of immune function and/or survival [47]. Interestingly, random site effects (e.g., site size, nesting substrate, type of predator fencing, monitoring frequency) explained negligible amounts of the variance in clutch size and fledgling productivity. The absence of large random site effects suggests the mechanisms shaping variation in clutch size and productivity operate at broad geographic scales.

### Predation and abandonment

Predation and abandonment can play a conspicuous role in limiting productivity but may also play an inconspicuous role in shaping clutch size estimates. As a result, it is important to consider whether they could potentially bias inferences in this study. I used clutch size estimates that were generated by averaging data from first clutches and from clutches laid to replace depredated or abandoned nests. If replacement clutches are smaller than first clutches in California least tern, it is conceivable that small average clutch sizes at a particular site could result from high egg predation or abandonment rates and large numbers of replacement clutches rather than reduced reproductive investment.

This scenario, which suggests a potential source of bias in clutch size estimates, seems unlikely, however, as replacement clutches are the same size as first clutches in common terns [48]. Furthermore, even should some bias due to predation exist, it seems unlikely that predators could generate consistent patterns of clutch size variation seen throughout the state from 1988 to 2009 (Figure 2). Predation rates would have had to increase at nearly every site from 1990 to 2004 then decline at nearly every site from 2004 to 2009 even while population size increased then leveled off over the same intervals (Figure 1). It is much easier to imagine abandonment rates varying in concert across the breeding range. However, if abandonment rates influenced clutch size estimates then the three hypotheses described above could still be invoked and tested as reasonable explanations for variation in abandonment rates.

### Management and policy implications

These analyses reveal significant temporal and spatial patterning in key demographic parameters, but they also highlight significant gaps in our understanding of the mechanisms that produce those patterns. The causes of long-term declines in clutch size and productivity, in particular, should be scrutinized despite population growth. Each of the three hypotheses I outline could account for the declines and, depending on which is/are supported or falsified, could lead to very different perspectives on the success of population recovery and the priorities for future management.

If the prey base of California least terns is shrinking then continued population growth may be severely limited or reversed until prey can be managed at broad geographic scales. If density-dependent competition has dictated variation in clutch size and productivity, the priority of future management should be to determine whether sufficient breeding opportunities are currently available to insure long-term recovery or whether additional sites are required to buffer the population from extinction. If the declines emerged as a result of age-dependent clutch size and productivity coupled with a shifting population age-structure, neither measure may be particularly amenable to management and there may be less concern about an ongoing recovery. Until the mechanistic links between demographic parameters and population change are described, however, the success of past management and the probability of ongoing recovery will remain difficult to characterize, as will the status of California least tern.

## References

[1] Beissinger SR, Westphal MI (1998). On the use of demographic models of population viability in endangered species management.. J Wildlife Manage.

[2] Caswell H (2000). Matrix population models: construction, analysis, and interpretation.

[3] US Fish and Wildlife Service (1985). Recovery Plan for the California least tern, *Sterna antillarum browni*.

[4] Chambers WL (1908). The present status of the Least Tern in southern California.. Condor.

[5] Lamb CC (1922). A unique colony of Least Terns.. Condor.

[6] US Fish and Wildlife Service (2006). California least tern, *Sternula antillarum browni*, 5-year review summary and evaluation.

[7] Marschalek DA (2010). California least tern breeding survey, 2009 season..

[8] Atwood JL, Kelly PR (1984). Fish dropped on breeding colonies as indicators of least tern food habits.. Wilson Bull.

[9] Massey BW, Bradley DW, Atwood JL (1992). Demography of a California Least Tern colony including effects of the 1982–1983 El Niño.. Condor.

[10] Moreau RE (1944). Clutch size: a comparative study with special reference to African birds.. Ibis.

[11] Lack D (1947). The significance of clutch size.. Ibis.

[12] Massey BW, Atwood JL (1981). Second-wave nesting of the California Least Tern: age composition and reproductive success.. Auk.

[13] Thompson BC, Jackson JA, Burger J, Hill LA, Kirsch EM, Poole A (1997). Least Tern (*Sterna antillarum*).. The Birds of North America Online.

[14] Collins CT, Massey BW, Dares LM, Wimer M, Gazzinga K (1996). Banding of adult California Least Terns at Marine Corps Base, Camp Pendleton between 1987 and 1995..

[15] Massey BW (1973). Recoveries of California Least Terns.. West Bird Bander.

[16] Smith TM, Reynolds RW, Peterson TC, Lawrimore J (2008). Improvements to NOAA's Historical Merged Land-Ocean Surface Temperature Analysis (1880–2006).. J Climate.

[17] Climate Prediction Center website.. http://www.cpc.ncep.noaa.gov/products/analysis_monitoring/ensostuff/ensoyears.shtml.

[18] Xue Y, Smith TM, Reynolds RW (2003). Interdecadal changes of 30-yr SST normals during 1871–2000.. J Climate.

[19] Bates D, Maechler M (2010). lme4: Linear mixed-effects models using S4 classes.. http://CRAN.Rproject.org/package=lme4.

[20] R Development Core Team (2010). R: A language and environment for statistical computing.. http://www.R-project.org.

[21] Zuur AF, Ieno EN, Walker NJ, Saveliev AA, Smith GM (2009). Mixed effects models and extensions in ecology with R.

[22] Wood SN (2004). Stable and efficient multiple smoothing parameter estimation for generalized additive models.. J Am Stat Assoc.

[23] Wood SN (2008). Fast stable direct fitting and smoothness selection for generalized additive models.. J Roy Stat Soc B.

[24] Moore DJ, Williams TD, Morris RD (2000). Mate provisioning, nutritional requirements for egg production, and primary reproductive effort of female Common Terns, *Sterna hirundo*.. J Avian Biol.

[25] Monaghan P, Uttley JD, Burns MD, Thaine C, Blackwood J (1989). The relationship between food supply, reproductive effort and breeding success in Arctic Terns *Sterna paradisaea*.. J Anim Ecol.

[26] Chavez FP, Ryan J, Lluch-Cota SE, Niquen MC (2003). From anchovies to sardines and back: multidecadal change in the Pacific Ocean.. Science.

[27] Robinette DP (2004). Partitioning of food resources by four sympatric terns (Aves: Laridae) breeding in southern California..

[28] Elliott ML, Hurt R, Sydeman WJ (2007). Breeding Biology and Status of the California Least Tern *Sterna antillarum browni* at Alameda Point, San Francisco Bay, California.. Waterbirds.

[29] Ashmole NP (1963). The regulation of numbers of tropical oceanic birds.. Ibis.

[30] Both C, Tinbergen JM, Visser ME (2000). Adaptive density dependence of avian clutch size.. Ecology.

[31] Birt VL, Birt TP, Goulet D, Cairnse DK, Montevecchi WA (1987). Ashmole's halo: direct evidence for prey depletion by a seabird.. Mar Ecol-Prog Ser.

[32] Lewis S, Grémillet D, Daunt F, Ryan PG, Crawford RJM (2006). Using behavioural and state variables to identify proximate causes of population change in a seabird.. Oecologia.

[33] Anderson SK, Roby DD, Lyons DE, Collis K (2007). Relationship of Caspian tern foraging ecology to nesting success in the Columbia River estuary, Oregon, USA.. Estuar Coast Shelf S.

[34] Lewis S, Sherratt TN, Hamer KC, Wanless S (2001). Evidence of intra-specific competition for food in a pelagic seabird.. Nature.

[35] Ballance LT, Ainley DG, Ballard G, Barton K (2009). An energetic correlate between colony size and foraging effort in seabirds, an example of the Adélie penguin, *Pygoscelis adeliae*.. J Avian Biol.

[36] Atwood JL, Minsky DE (1983). Least tern foraging ecology at three major California breeding colonies.. Western Birds.

[37] Nisbet ICT, Apanius V, Friar MS (2002). Breeding performance of very old common terns.. J Field Ornithol.

[38] Rebke M, Coulson T, Becker PH, Vaupel JW (2010). Reproductive improvement and senescence in a long-lived bird.. P Natl Acad Sci USA.

[39] Galbraith H, Hatch JJ, Nisbet ICT, Kunz TH (1999). Age-related changes in efficiency among breeding common terns *Sterna hirundo*: measurement of energy expenditure using doubly-labelled water.. J Avian Biol.

[40] Limmer B, Becker PH (2009). Improvement in chick provisioning with parental experience in a seabird.. Anim Behav.

[41] Collins CT, Baird PH, Bradley RA (1990). Banding of adult California Least Terns at Camp Pendleton Marine Base 1987–1990: Summary Report..

[42] Haussmann MF, Mauck RA (2008). New strategies for telomere-based age estimation.. Mol Ecol Resour.

[43] Haussmann MF, Vleck CF, Nisbet ICT (2003). Calibrating the telomere clock in common terns, *Sterna hirundo*.. Exp Gerontol.

[44] Horn T, Robertson BC, Gemmell NJ (2010). The use of telomere length in ecology and evolutionary biology.. Heredity.

[45] Cane MA (2005). The evolution of El Niño, past and future.. Earth Planet Sc Lett.

[46] Winkler DW, Walters JR (1983). The determination of clutch size in precocial birds.. Curr Ornithol.

[47] Ardia DR (2005). Tree swallows trade off immune function and reproductive effort differently across their range.. Ecology.

[48] Wendeln H, Becker PH, González-Solís J (2000). Parental care of replacement clutches in common terns (*Sterna hirundo*).. Behav Ecol Sociobiol.

